# The brand-new predictor index of fulminant process in patients with acute myocarditis: hemoglobin, albumin, lymphocyte and platelet (HALP) score

**DOI:** 10.3389/fnut.2025.1674368

**Published:** 2025-10-29

**Authors:** Pelin Aladag, Ahmet Avci

**Affiliations:** Department of Cardiology, Bulent Ecevit University Faculty of Medicine, Zonguldak, Türkiye

**Keywords:** HALP score, acute myocarditis, fulminant myocarditis, immunonutrion, biomarker, prognostic

## Abstract

**Background/objectives:**

Myocarditis can progress to a fulminant form, leading to severe heart failure and mortality. Inflammation and nutritional status play critical roles in disease progression. The hemoglobin, albumin, lymphocyte, and platelet (HALP) score is a novel, easily accessible biomarker that reflects both systemic inflammation and nutritional status. This study aimed to evaluate the prognostic value of the HALP score in predicting fulminant myocarditis among patients diagnosed with acute myocarditis.

**Methods:**

A total of 124 patients diagnosed with acute myocarditis were retrospectively enrolled in this single-center study. Patients were categorized into non-fulminant and fulminant myocarditis groups based on diagnostic criteria.

**Results:**

The median age of the patients was 24 years, 14.5% was female. Twenty-three of these patients (18.5%) exhibited fulminant myocarditis. Troponin I, C-reactive protein (CRP), white blood cell (WBC), and N-terminal pro-brain natriuretic peptide (NT-proBNP) levels were significantly higher in the fulminant myocarditis group, while the HALP score was lower (*p* < 0.001 for all). Multivariable regression analysis identified WBC, NT-proBNP and HALP score as independent predictors of fulminant myocarditis (*p* = 0.029, *p* = 0.011 and *p* = 0.046, respectively). The optimal cut-off value of the HALP score was 4.12 for predicting fulminant myocarditis, with an area under the curve of 0.814. Beyond its diagnostic utility, a low HALP score was also significantly associated with worse clinical outcomes.

**Conclusion:**

The HALP score can independently predict the development of fulminant myocarditis in acute myocarditis patients.

## 1 Introduction

Myocarditis is defined as an inflammatory infiltrative disease of the myocardium; it is caused by infective or non-infective components, viruses are the most common cause (1, 2). In the literature, the prevalence of myocarditis is approximately 4–14 per 100,000 people globally, with a mortality rate of approximately 1–7% (3).

Myocarditis is usually found in young individuals. The clinical presentation of myocarditis has a wide spectrum; it may be completely asymptomatic or progress to severe cardiac failure, resulting in death (4). Within 2 weeks following the viral prodrome, the patient may progress to fulminant myocarditis, in which symptoms of advanced heart failure are present, requiring inotropic therapy, ventricular assist devices to support cardiogenic shock or emergency treatment of fatal severe arrhythmias (5). Therefore, the use and identification of effective and appropriate prognostic biomarkers is critical for the early diagnoses of high-risk patients for fulminant myocarditis and the accurate medical treatment management.

In recent years, The haemoglobin, albumin, lymphocyte, and platelet (HALP) score is a novel predictive indicator evaluating patient prognosis in many diseases (6, 7). Each component of the HALP score provides important information about inflammatory and nutritional processes. Anemia is an important indicator of severe inflammatory processes, as assessed by measuring hemoglobin levels (8). Albumin, which is both a nutritional marker and a negative acute phase reactive protein that reflects the immunonutritional status (9). Low lymphocyte and high platelet levels, which are fundamental components of immune function, indicate immune dysfunction and an increased risk of infection (10). Given the importance of inflammatory and nutritional parameters in predicting patient prognosis and performing risk classification, the HALP score, which combines these factors for assessment, shows significant potential.

In this context, the HALP score has been shown to be an important independent predictor of disease severity and inflammation in major cardiac diseases such as coronary artery disease and heart failure (7, 11, 12). Despite strong evidence for the prognostic value of the HALP score in patients with heart disease, no study in the literature has evaluated the HALP score in patients with acute myocarditis.

The aim of this study was to evaluate the effectiveness of the HALP score in the prediction of fulminant myocarditis and to quantify its prognostic value in patients with acute myocarditis.

## 2 Materials and methods

### 2.1 Study design and population

The study was designed as a retrospective cross sectional and included patients aged 18 years and older who were hospitalized in the emergency department or outpatient clinic of Zonguldak Bülent Ecevit University Hospital between January 2014 and January 2025 with a diagnosis of acute myocarditis and who had no previous history of heart disease. Patients with structural heart disease, coronary artery disease, inflammatory or autoimmune diseases, leukemia or other blood system diseases, malignancies, pregnancy, and missing medical data in the hospital registry system were excluded from the study.

### 2.2 Definitions

The current diagnostic criteria for myocarditis include the signs and symptoms of acute cardiac dysfunction (e.g., dyspnoea, syncope, exercise intolerance, chest pain, tachypnoea, tachycardia, gallop rhythm), elevated troponin value, echocardiographic (ECHO) evidence of ventricular dysfunction, presence of prodromic respiratory or gastrointestinal infection diseases within 2 weeks of symptom onset, specific electrocardiogram (ECG) changes (13). Based on the latest guidelines, ECG abnormalities were accepted as sinus tachycardia, supraventricular tachycardia, ventricular arrhythmia, conduction abnormalities and ST segment changes (13).

Fulminant and non-fulminant myocarditis patients were categorized into two groups. The fulminant progression of acute myocarditis is defined as patients with a low ejection fraction (<40%) and severe haemodynamic compromise requiring inotropic therapy or ventricular assist devices, the development of end-organ failure (14).

### 2.3 Data collection

Data related to demographic characteristics, vital signs, comorbidities, laboratory values, length of stay, patient management and in-hospital complications in the coronary care unit (CCU), mortality, ECG and ECHO findings were enrolled from the hospital system. All patients recorded in the study was screened with transthoracic ECHO using Philips Affiniti 50 ultrasound device (Philips Healthcare, Amsterdam, The Netherlands) and S5-1 transthoracic probe (Philips Healthcare, Amsterdam, The Netherlands) by a cardiologist. Left ventricular end-diastolic diameter, valve pathologies and pericardial effusion were assessed from parasternal and apical views. The left ventricular ejection fraction (LVEF) was measured by the Simpson’s method.

Laboratory values were based on blood samples received during the first medical evaluation immediately after admission to the emergency department or outpatient clinic. Laboratory assessments include white blood cell (WBC), hemoglobin, albumin, absolute lymphocyte count (ALC), absolute neutrophil count (ANC), platelet count (PLT), albumin levels, C-reactive protein (CRP), serum troponin and N-terminal pro-brain natriuretic peptide (NT-proBNP) levels and other biochemical tests. The HALP score was first defined by Chen et al. as follows (15):


$$\text{HALP}=\frac{\text{Lymphocyte count}\times \text{Albumin}\times \text{Hemoglobin}\;}{\text{Platelet count}\;}$$

### 2.4 Outcomes

The primary outcome is to compare the HALP score of patients with fulminant and non-fulminant myocarditis and to analyse other markers of both groups and to identify other factors influencing the prognosis of myocarditis. The second outcome is the predictive power of the HALP score on prognosis.

### 2.5 Statistical analysis

The Kolmogorov–Smirnov test is used to assess the normality of the data in a numerical variable. Normal distributed values are shown as mean with standard deviation and non-normally distributed variables are represented as median with interquartile intervals. For inter-group comparisons, categorical variables were statistically analysed using the chi square test and variables were defined as percentages (%). Independent samples t-tests or Mann–Whitney U tests were used to compare two groups of continuous variables. Spearman’s correlation was used to analyse the correlation between HALP score and LVEF. To determine independent predictors of fulminant myocarditis, variables demonstrating significance in univariate analysis (*p* < 0.10) were sampled for inclusion in multivariate logistic regression models. Backward LR method was used in all regression models. Receiver operating characteristic (ROC) analysis was applied to determine the optimal cut-off value of HALP score in prediction of fulminant myocarditis. The area under the curve (AUC) was calculated to predict fulminant myocarditis for the HALP score and other markers.

Data analysis was conducted using the Statistical Package for the Social Sciences 26.0 (SPSS Inc., IL, USA). A statistically significant difference was defined as two-tailed *p* < 0.05.

## 3 Results

**Figure 1:**
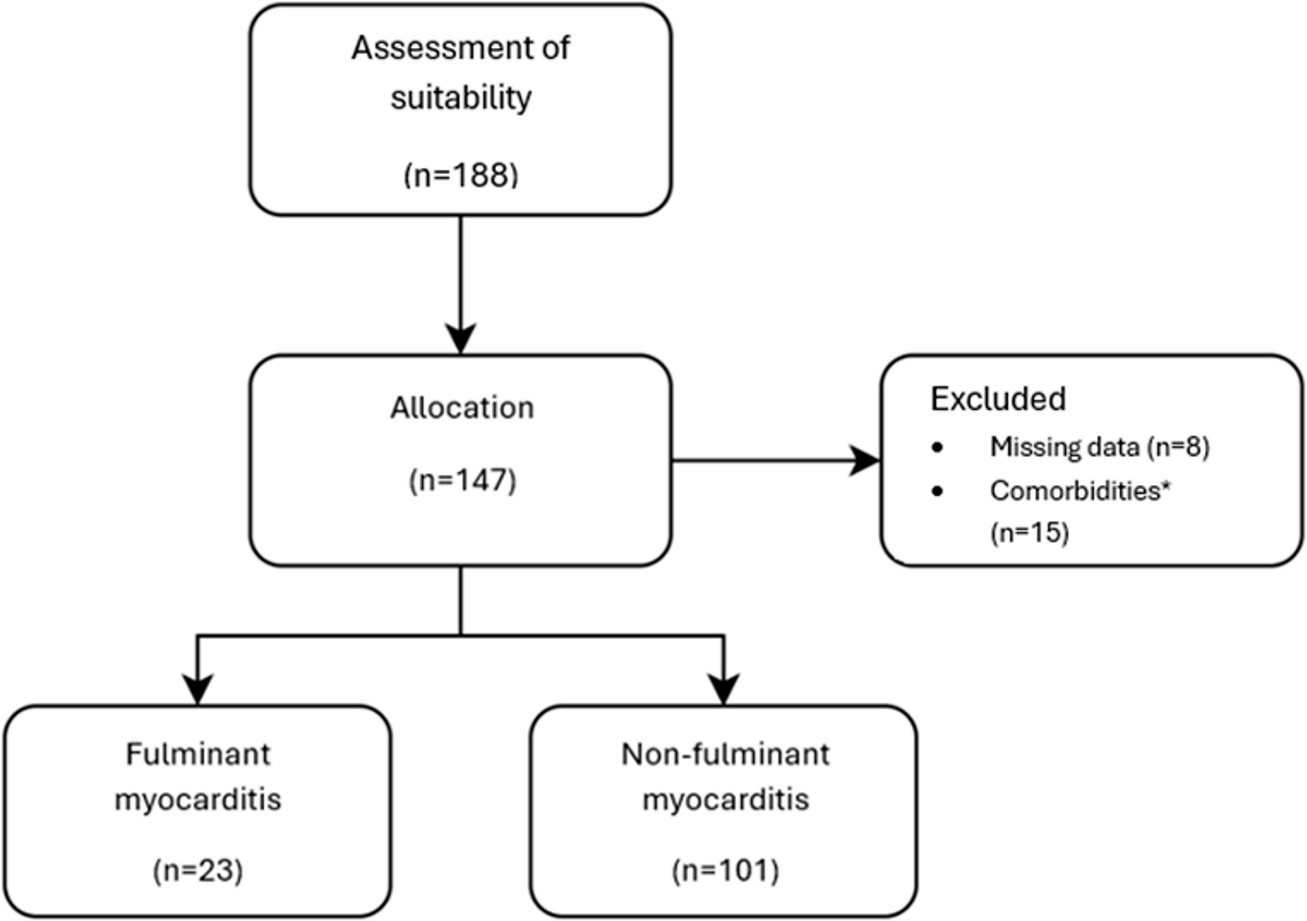
Flow chart of patients. *Patients with prior CAG and PTCA history, malignancy history, pre-existing HF history, blood system disease including essential thrombocytosis, cardiac transplant, autoimmune disease including systemic lupus erythematosus, cancer, leukemia.

One hundred and eighty-six adult patients were diagnosed with acute myocarditis in the emergency department, and a total of 124 patients were enrolled in the study. The flow chart of the enrolment process is given in Figure 1.

Of the patients enrolled in the study, 106 (85.4%) were male and the median age was 24 (19–32) years. Fulminant myocarditis was diagnosed in 23 (18.5%) of the patients. There were no significant differences between the groups regarding age, gender, or other demographic characteristics (*p* > 0.050 for all). All characteristics of all patients with fulminant myocarditis and non-fulminant myocarditis including demographic data, clinical follow-up data and outcomes are shown in Tables 1, 2.

**Table 1:** Distribution of baseline demographics, laboratory, ECG and echocardiographic parameters, and clinical follow-up data in the fulminant and non-fulminant groups.

Variables	Non-fulminant group (*n* = 101)	Fulminant group (*n* = 23)	*p* value
Age, years (median [IQR])	24 [20–27]	23 [19–32]	0.7
Male, [*n* (%)]	85 (84.2%)	21 (91.3%)	0.52
Hypertension, [*n* (%)]	4 (4%)	1 (4.3%)	0.93
Diabetes mellitus, [*n* (%)]	7 (6.9%)	3 (13%)	0.39
ECG and echocardiographic variables			
ECG abnormalities, [*n* (%)]	8 (7.9%)	5 (21.7%)	0.06
Pericardial effusion, [*n* (%)]	22 (21.8%)	23 (100%)	<0.001
LVEF, [*n* (%)]	60 [55–65]	30 [20–35]	<0.001
NYHA class [*n* (%)]			
NYHA class I	46 (45.5%)	0	<0.001
NYHA class II	49 (48.5%)	3 (13%)	0.002
NYHA class III	6 (5.9%)	5 (21.7%)	0.03
NYHA class IV	0	15 (65.2%)	<0.001
Laboratory parameters (median [IQR])			
Creatinine (mg/dL)	0.85 [0.7–0.99]	1.14 [0.9–1.4]	<0.001
ALT (U/L)	24 [19–34]	57 [31–516]	<0.001
AST (U/L)	28 [21–37]	118 [40–589]	<0.001
LDH (U/L)	313 [224–387]	642.5 [349–887.7]	<0.001
Troponin I (ng/mL)	0.78 [0.19–3.4]	5.73 [1.34–24.2]	<0.001
NT-proBNP (pg/mL)	109 [82–212.4]	984 [522.5–1,675]	<0.001
CRP (mg/L)	21.4 [12–39.7]	64.1 [35.3–100.2]	<0.001
Procalcitonin (ng/mL)	0.09 [0.06–0.4]	1.44 [0.99–2.1]	<0.001
WBC (×109/L)	8.03 [6.15–9.6]	10.2 [7.9−16.9]	<0.001
ANC (×109/L)	4.55 [3.4–6.44]	8.5 [5.37–14.5]	<0.001
ALC (×109/L)	2.02 [1.55–2.7]	1.47 [1.1–1.9]	0.001
Platelet (×109/L)	236 [203–299]	271 [213–320]	0.14
Hemoglobin, g/DL	15.1 [14.3–15.7]	14.2 [12.6–15.1]	0.004
Albumin, g/dL	44 [43–47]	42 [39–45]	<0.001
HALP score	5.69 [4.1–7.66]	2.7 [2.18–4.65]	<0.001

This table compares various parameters between patients with fulminant myocarditis (*n* = 23) and non-fulminant myocarditis (*n* = 101). Parameters include demographic data, laboratory parameters, ECG and echocardiographic variables. *p*-values indicate statistical significance between the two groups. ALC, absolute lymphocyte count; ALT, alanine aminotransferase; AST, aspartate aminotransferase; ANC, absolute neutrophil count; CRP, C-reactive protein; ECG, electrocardiogram; ESR, erythrocyte sedimentation rate; HALP score, the hemoglobin, albumin, lymphocyte, and platelet score; LDH, lactate dehydrogenase; LVEF, left ventricular ejection fraction; NT-proBNP, N-terminal pro-brain natriuretic peptide; NYHA, New York Heart Association; WBC, white blood cell count.

**Table 2:** Comparison of clinical outcomes in the fulminant and non-fulminant groups.

Variables	Non-fulminant group (*n* = 101)	Fulminant group (*n* = 23)	*p* value
Mechanical ventilation, [*n* (%)]	0	6(26.1%)	<0.001
Length of stay in CCU (day)	0(0–2)	5(1–13)	<0.001
CCU admission, [*n* (%)]	50(49.5%)	23(100%)	<0.001
Inotropes, [*n* (%)]	8(7.9%)	23(100%)	<0.001
Complication^a^, [*n* (%)]	5(5%)	11(47.8%)	<0.001
In-hospital mortality, [*n* (%)]	0	6(26.1%)	<0.001

^a^Dilated cardiomyopathy, malignant arrhythmia, cardiac tamponade, renal failure requiring hemodialysis. This table compares clinical follow-up data between patients with fulminant myocarditis (*n* = 23) and non-fulminant myocarditis (*n* = 101). *p*-values indicate statistical significance between the two groups. CCU: Coronary care unit.

ECG abnormalities were detected in 8 (7.9%) non-fulminant patients and 5 (21.7%) fulminant patients. There was no significant difference in ECG abnormalities between the two cohorts (*p* = 0.060). The median value of LVEF was significantly lower in the fulminant myocarditis group (30% [25–35]) than in the non-fulminant myocarditis group (60% [55–65]) (*p* < 0.001). Pericardial effusion was found in all patients with fulminant myocarditis and in 22 (21.7%) non-fulminant patients, and there was a significant difference between the groups (*p* < 0.001).

The median HALP level was 2.7 [2.18–4.65] in the fulminant myocarditis group and 5.69 [4.1–7.66] in the non-fulminant group, and there was a significant difference between the groups (*p* < 0.001). There was significant difference between the groups in cardiac markers. The Troponin I level in the fulminant myocarditis group (5.73 ng/mL [1.34–24.2]) was significantly higher than that in the non-fulminant myocarditis group (0.78 ng/mL [0.19–3.4]) (*p* < 0.001). The median NT-proBNP level was significantly higher in the fulminant myocarditis group (984.0 pg./mL [522.5–1675.0]) than in the non-fulminant myocarditis group (109.0 pg./mL [82.0–212.4]) (*p* < 0.001). Similarly, median WBC, ANC, CRP and procalcitonin values were higher in the fulminant myocarditis cohort (*p* < 0.001, for all). In contrast, the median ALC, hemoglobin and albumin were significantly lower in the fulminant myocarditis group than in the non-fulminant myocarditis group (*p* < 0.001, *p* = 0.004 and *p* < 0.001, respectively). There was no significant difference between the groups in terms of the median platelet counts (*p* = 0.140).

Univariate logistic analysis showed that WBC, NT-proBNP, Troponin I, CRP, albumin, hemoglobin levels and HALP score could predict fulminant myocarditis. According to the results of multivariate logistic regression, that WBC, NT-proBNP values and HALP score were independent parameters that could be used to differentiate between the fulminant myocarditis and non-fulminant myocarditis groups (*p* = 0.029, *p* = 0.011 and *p* = 0.046, respectively), but not troponin I and CRP (*p* = 0.059 and *p* = 0.356), as demonstrated in Table 3.

**Table 3:** Independent predictors of fulminant myocarditis according to the multiple logistic regression analysis.

Characteristics	Univariable analysis		Multivariable analysis	
	OR (95%CI)	*p*-value	aOR (95% CI)	*p*-value
WBC	1.375(1.172–1.613)	<0.001	1.923(1.069–3.458)	0.029
NT-proBNP	1.006(1.004–1.009)	<0.001	1.006(1.001–1.010)	0.011
HALP score	0.540(0.395–0.738)	<0.001	0.379(0.146–0.983)	0.046
CRP	1.024(1.011–1.036)	<0.001	1.022(0.976–1.071)	0.356
Albumin	0.755(0.643–0.886)	0.001	0.741(0.467–1.177)	0.204
Hemoglobin	0.561(0.387–0.814)	0.002	0.482(0.189–1.225)	0.125
Troponin I	1.161(1.085–1.243)	<0.001	1.208(0.993–1.470)	0.059
Platelet	1.005(0.999–1.011)	0.107	_	_

This table shows the adjusted odds ratios (aOR) with 95% confidence intervals (CI) and *p*-values for the predictors of fulminant myocarditis. CRP, C-reactive protein; HALP score, the hemoglobin, albumin, lymphocyte, and platelet score; NT-proBNP, N-terminal pro-brain natriuretic peptide; WBC, white blood cell count.

Spearman’s correlation analysis showed a significant positive correlation between HALP score and LVEF (*r* = 0.421, *p* < 0.001).

**Figure 2:**
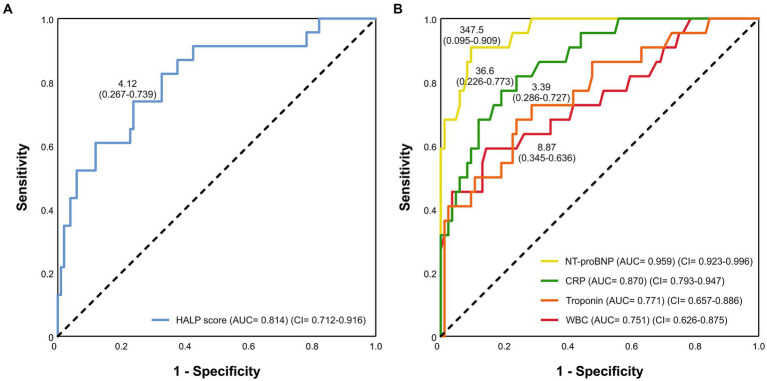
The ROC curve was used to evaluate the **(A)** HALP score and **(B)** other biomarkers for predicting fulminant myocarditis.

In ROC analysis, the HALP score predicted fulminant myocarditis (95% confidence interval [CI]: 0.712–0.916, *p* < 0.001). The AUC was 0.814. The best cut-off value was 4.12, and the sensitivity was 73.9% and the specificity was 73.3% (Figure 2). In order to assess the discriminatory ability of other biomarkers between the two groups, ROC analysis was also performed for these markers, (*p* < 0.001, for all). The results of this analysis are shown in Figure 2. The *p*-values associated with the AUC values confirm the statistical significance of the results, thereby providing evidence that the predictive ability of these biomarkers is also reliable. The AUC values for NT-proBNP, CRP, and HALP score are all greater than 0.8, indicating that they have strong discriminatory power.

The cohort were divided into two groups on the basis of a HALP score with a cut-off value of 4.12. The CCU admission occurred in patients 39 (90.7%) with HALP score <4.12, of whom 23 cases received inotropes, 6 cases required mechanical ventilation, 13 cases had complications, and 6 cases died. The CCU admission rates were higher in patients with the HALP score < 4.12 (29.8%) compared with those with the HALP score ≥4.12 (56.2%) (*p* < 0.001). Patients with the HALP score <4.12 had a longer CCU stay than the other group (*p* < 0.001). The incidence of complications was significantly higher in the HALP score <4.12 (*p* < 0.001). In addition, none of the patients with a HALP score ≥4.12 had the need of invasive mechanical ventilation, and no in-hospital mortality was observed in these patients (Table 4).

**Table 4:** The comparison of clinical results between patients with HALP score <4.12 and HALP score ≥4.12.

Clinical data	HALP <4.12 (*n* = 43)	HALP ≥4.12 (*n* = 81)	*p* value
Mechanical ventilation, [*n* (%)]	6(14%)	0	0.001
Length of stay in CCU (day)	2(0–5)	0(0–1)	<0.001
CCU admission, [*n* (%)]	39(90.7%)	34(42%)	<0.001
Inotropes, [*n* (%)]	23(53.5%)	8(9.9%)	<0.001
Complication^a^, [*n* (%)]	13(30.2%)	3(3.7%)	<0.001
In-hospital mortality, [*n* (%)]	6(14%)	0	0.001

^a^Dilated cardiomyopathy, malignant arrhythmia, cardiac tamponade, renal failure requiring hemodialysis. This table compares clinical data between patients with an HALP score <4.12 (*n* = 43) and those with an HALP score of 4.12 or greater (*n* = 81). Outcomes include CCU admission, length of stay in CCU, use of inotropes, in-hospital mortality, complications and mechanical ventilation. *p*-values indicate statistical significance. CCU: Coronary care unit.

## 4 Discussion

This study is the first to investigate the association of the HALP score with disease prognosis and clinical outcomes in acute myocarditis patients. The current study showed that in patients with a diagnosis of acute myocarditis, the HALP score was independently associated with a significant likelihood of a fulminant course of disease. In our study, the prognostic value of the HALP score in patients with acute myocarditis was also investigated, patients exhibiting a HALP cut-off value below 4.12 were found to have a significantly higher risk of developing fulminant myocarditis. Furthermore, a low HALP score was found to be an effective predictor of CCU admission, the necessity of mechanical ventilation, the requirement of inotropes, and mortality in patients with myocarditis. For these reasons, it may be appropriate to closely monitor patients with values below this threshold in daily practice and to evaluate them in detail in terms of their need for intensive care. Its implementation in clinical practice facilitates expeditious identification of high-risk patients, thereby enabling more informed and rapid medical decision-making, potentially driving more personalized treatment strategies.

The HALP score is derived from four routine laboratory parameters: hemoglobin, albumin, lymphocyte count, and platelet count (12). Low hemoglobin and albumin levels reflect anemia and malnutrition, while decreased lymphocyte and elevated platelet counts indicate immune dysregulation and systemic inflammation (16). By integrating these components, the HALP score serves as a comprehensive index of both nutritional and inflammatory status (17). Previous studies have demonstrated its prognostic utility in various clinical conditions, including malignancies and cardiovascular diseases (6, 17). Notably, lower HALP scores have been associated with in-hospital mortality in patients with non-ST-elevation myocardial infarction and with poor outcomes such as no-reflow and major adverse cardiovascular events in ST-elevation myocardial infarction (18, 19).

Each component of the HALP score has been individually associated with disease severity in cardiovascular conditions. Low albumin levels have been linked to worse outcomes in acute myocarditis, while chronic anemia contributes to ventricular remodeling and increased cardiovascular mortality risk (8, 20, 21). Elevated platelet counts have been correlated with poor short-term outcomes in acute myocardial infarction, and lymphopenia is a recognized marker of systemic inflammation (22). New systemic inflammatory indices, including lymphocyte count, provide a relevant measure of the severity of the inflammatory response. This response has been shown to play an important role in the progression of acute myocarditis to a fulminant process (23). In a recent study, Erbay et al. identified the systemic immune-inflammation index (SII) as an independent predictor of fulminant myocarditis (24). In studies conducted by Eyiol et al. Hemoglobin/Red Blood Cell Distribution Width Ratio (HRR) and Red Blood Cell Distribution Width/Albumin Ratio (RAR) parameters, which assess nutritional and inflammatory status, have been shown to be biomarkers that predict the severity and prognosis of myocarditis. It has been emphasized that, in contrast to conventional markers, providing additional information related to prognosis contributes to the personalization of treatment management (9, 25).

Therefore, our findings suggest that the HALP score may offer a broader and more integrative perspective, as it captures both systemic inflammation and nutritional status. This dual capacity highlights the HALP score as a potentially superior biomarker for prognostic stratification in patients with acute myocarditis.

Myocardial necrosis is a key feature of fulminant myocarditis and typically leads to a significant rise in cardiac troponin levels. Although elevated troponin I levels were observed in patients with fulminant myocarditis in our study, this marker did not remain an independent predictor in multivariable analysis (26). In contrast, Freixa et al. previously reported that normal or only mildly elevated troponin I levels on admission may paradoxically indicate a worse prognosis in fulminant cases, possibly reflecting extensive and rapid myocardial injury with limited biomarker release (27). In this study, troponin I levels of patients with fulminant myocarditis were significantly higher than those of patients with non-fulminant myocarditis but were not demonstrated to be a significant independent predictor.

Similarly, CRP and platelet counts did not emerge as independent predictors in our analysis, despite being elevated in fulminant myocarditis. This finding is consistent with prior studies that reported no significant association between CRP levels and fulminant progression (28, 29). In contrast, WBC count was identified as an independent prognostic marker. Given that WBC reflects the combined effect of neutrophilia and lymphopenia, its elevation in fulminant myocarditis is biologically plausible and aligns with previous reports (28–30). Nevertheless, our study aimed to go beyond conventional inflammatory markers and identify a more comprehensive prognostic tool, leading to the evaluation of the HALP score.

In our study, NT-proBNP levels were significantly higher in patients with fulminant myocarditis, and this biomarker emerged as an independent predictor of disease severity. Previous research, including the study by Gassan et al. has suggested that elevated brain natriuretic peptide (BNP) levels in acute myocarditis result from neurohormonal activation triggered by myocardial inflammation and oedema-induced wall stress (31). Although the prognostic utility of NT-proBNP in myocarditis is well established, its routine use may be limited by practical factors such as cost, availability, and its primary role as a heart failure marker rather than a specific indicator of myocarditis-related deterioration.

### 4.1 Limitations

This study has several limitations. Its retrospective and single-center design may limit the generalizability of the findings. The relatively small number of patients with fulminant myocarditis could reduce the statistical robustness of subgroup analyses. HALP scores were calculated only at the time of initial admission, and changes in these values during the course of hospitalization were not assessed. In addition, although endomyocardial biopsy and cardiac magnetic resonance imaging are considered the gold standards for diagnosing myocarditis, most patients in this cohort were diagnosed clinically due to limited access to these advanced modalities.

## 5 Conclusion

The HALP score appears to be an independent predictor of fulminant progression in patients with acute myocarditis. As a simple and accessible composite marker that reflects both inflammatory and nutritional status, the HALP score may help identify high-risk patients at an early stage and guide clinical decision-making. The studies included multicentred and larger sample populations are needed to further validate our outcomes.

## Data Availability

The raw data supporting the conclusions of this article will be made available by the authors, without undue reservation.
